# *Klebsiella* in Wildlife: Clonal Dynamics and Antibiotic Resistance Profiles, a Systematic Review

**DOI:** 10.3390/pathogens13110945

**Published:** 2024-10-30

**Authors:** Micaela Quintelas, Vanessa Silva, Sara Araújo, Maria Teresa Tejedor-Junco, José Eduardo Pereira, Gilberto Igrejas, Patricia Poeta

**Affiliations:** 1 Microbiology and Antibiotic Resistance Team (MicroART), Department of Veterinary Sciences, University of Trás-os-Montes and Alto Douro (UTAD), 5000-801 Vila Real, Portugalsaravanessaaraujo07@gmail.com (S.A.); jeduardo@utad.pt (J.E.P.); 2 LAQV-REQUIMTE, Department of Chemistry, NOVA School of Science and Technology, Universidade Nova de Lisboa, 2829-516 Caparica, Portugal; 3 Department of Genetics and Biotechnology, University of Trás-os-Montes and Alto Douro (UTAD), 5000-801 Vila Real, Portugal; 4 Functional Genomics and Proteomics Unit, University of Trás-os-Montes and Alto Douro (UTAD), 5000-801 Vila Real, Portugal; 5 Research Institute of Biomedical and Health Sciences, University of Las Palmas de Gran Canaria, 35001 Las Palmas de Gran Canaria, Spain; mariateresa.tejedor@ulpgc.es; 6 CECAV—Veterinary and Animal Research Centre, University of Trás-os-Montes and Alto Douro, 5000-801 Vila Real, Portugal; 7 Associate Laboratory for Animal and Veterinary Sciences (AL4AnimalS), University of Traás-os-Montes and Alto Douro (UTAD), 5000-801 Vila Real, Portugal

**Keywords:** *Klebsiella* spp., wild animals, multidrug resistance, One Health

## Abstract

*Klebsiella* spp. are a genus of Gram-negative, opportunistic bacteria frequently found in the flora of the mucosal membranes of healthy animals and humans, and in the environment. Species of this group can cause serious infections (meningitis, sepsis, bacteraemia, urinary tract infections, liver damage) and possible death in immunocompromised organisms (and even in immunocompetent ones in the case of hypervirulent *K. pneumoniae*) that are exposed to them. *K. pneumoniae* is part of the ESKAPE organisms, and so it is important to understand this genus in terms of multidrug-resistant bacteria and as a carrier of antibiotic resistance mechanisms. As it is a durable bacterium, it survives well even in hostile environments, making it possible to colonize all kinds of habitats, even the mucosal flora of wildlife. This systematic review explores the prevalence of *Klebsiella* spp. bacteria in wild animals, and the possibility of transmission to humans according to the One Health perspective. The isolates found in this review proved to be resistant to betalactams (*bla*_TEM_, *bla*_OXA-48_…), aminoglycosides (*strAB*, *aadA2*…), fosfomycin, tetracyclines, sulphonamides, trimethoprim, phenicols (catB4), and polymyxins (*mcr4*).

## 1. Introduction

Antibiotics are chemical compounds, natural or artificial, with antibacterial properties that can prevent the growth/proliferation of a bacterial culture or lead to its death. The properties of these drugs were discovered in 1928 by Alexander Fleming [1], through benzylpenicillin (penicillins), and first used en masse in 1935 and during World War II in 1942, in the cases of sulfonamides and penicillins, respectively [2].

However, some species of bacteria, which would later be affected by the use of these antibiotics, acquired resistance genes against them in the 1950s, thus becoming antibiotic-resistant bacteria [1,3]. In response, new beta-lactam antibiotics were developed, antibiotics that soon became obsolete in the treatment of bacterial cultures that became resistant [1,3], bringing back the threat of a return to a world without treatment for infectious diseases caused by bacteria. Such races between researchers and multiresistant bacteria are still happening, even in the modern age [4].

Antibiotics are considered one of the greatest medical discoveries, and the loss of their effectiveness against microbial substances can compromise the well-being of living things. Such a loss may occur due to overuse of these compounds, poor prescription (around 50% of antibiotics prescribed to patients are not needed), and poor planning of the time needed for the treatment [5,6,7], as well as due to the use of antibiotics in the agricultural industry both in the development of plant crops—the use of tetracyclines in fruit trees, for example—and in the formation of animal products—such as fattening and preventive medicine in farm animals. However, in the European Union, this use is highly regulated [8].

It has been proven that antibiotic-resistant bacteria are able to cross the species barrier when the same bacteria are found in the intestinal flora of both farmers and their animals [9], and that pathogenic bacteria do travel with the meat product to stores and, consequently, consumers [10]. Other obstacles are the low availability of new antibiotics and strict regulatory barriers that delay or hinder new studies [4]. International travel and low hygiene conditions potentiate the proliferation of bacteria and horizontal gene transfer (HGT) among them, as well as the release of antibiotic metabolites not processed by the body into the environment around it, which pushes the existing bacteria to evolve and adapt [1,3]. 

However, there is a natural component of antibiotic resistance that is millennia-old [11,12], that being the spread of antibiotic resistance genes by antibiotic producers [13], due to survival strategies, or by simple horizonal gene transfer. According to the One Health approach, which views the well-being of all ecosystems and those which inhabit them as being deeply connected and dependent on one another, the emergence and transfer of multiresistance genes from bacteria in the environment will eventually affect humans, and one route that possibly accelerates that result is coexistence with wildlife.

According to the World Health Organization (WHO), “One Health” is a unifying concept that aims to balance and improve the health of people, animals, and the environment through public health, veterinary medicine, and conservation of the environment. It is especially important when it comes to improving nutrition and food and water safety, to the fight against pollution, and to fighting antibiotic resistance and controlling zoonoses. The One Health perspective is a game-changing approach that focuses on the interdependence of animal, human, and environmental health to address global public health issues. This methodology becomes even more important when tackling antibiotic resistance due to the intricate web of connections within ecosystems. It is important to consider that, in wildlife environments, the presence of *Klebsiella* spp. and their antibiotic resistance may not solely be attributed to direct selective pressure from antibiotic exposure, which is more commonly associated with clinical or agricultural settings [14,15,16,17]. In these natural ecosystems, the selective pressures acting on microbial populations are more likely to be multifaceted, involving a combination of ecological and environmental factors, such as competition for resources, predation, host immune responses, and fluctuations in abiotic conditions (e.g., temperature, pH, nutrient availability). As such, the maintenance and proliferation of *Klebsiella* strains in wildlife may be influenced by broader fitness traits that allow these bacteria to survive and thrive in diverse and often harsh environments.

In addition to the well-characterized antibiotic resistance mechanisms, it is plausible that *Klebsiella* strains in wildlife rely on phenotypic plasticity—the ability to modify their phenotype in response to environmental changes—and non-specific fitness mechanisms. These could include enhanced biofilm formation, stress resistance pathways, or metabolic versatility, which allow the bacteria to adapt to a wide range of conditions beyond those related to antibiotic pressure [18,19]. Furthermore, the presence of antibiotic resistance genes in wildlife-associated *Klebsiella* strains could be driven by selective pressures unrelated to direct antibiotic exposure, such as the co-selection of resistance genes with other advantageous traits (e.g., heavy metal resistance) or their association with mobile genetic elements that carry multiple adaptive genes [20,21,22].

Understanding whether the selection pressure acting on *Klebsiella* strains is primarily due to antibiotic resistance genes or broader phenotypic plasticity mechanisms is crucial for unravelling the evolutionary dynamics of these bacteria in natural settings. This also raises the possibility that the observed resistance in wildlife may not solely be a result of human-driven antibiotic use but could also be linked to intrinsic survival strategies that predate anthropogenic impacts.

Future studies should focus on exploring the genetic basis of these non-specific fitness mechanisms in Klebsiella species isolated from wildlife. This could involve investigating the expression of genes related to environmental resilience, such as those involved in oxidative stress responses, quorum sensing, efflux pumps, and the capacity for horizontal gene transfer under natural environmental conditions. By integrating these aspects, future research can provide a more comprehensive understanding of the factors driving the persistence and dissemination of antibiotic resistance in wildlife microbiomes, as well as the role that these ecosystems play in the global spread of resistant pathogens.

Given the increasing interface between wildlife, humans, and domestic animals, particularly in the context of habitat encroachment and climate change, addressing these questions is critical. Wildlife ecosystems may serve as reservoirs not only for antibiotic-resistant bacteria but also for novel resistance mechanisms that could emerge through genetic exchange and environmental pressures. As such, this perspective should be considered in efforts to mitigate the global antibiotic resistance crisis.

Throughout the world, there is a wide diversity of wild animals in various environments ranging from birds and fish to mammals. These environments also shelter bacteria, including dangerous, pathogenic species that are resistant to antibiotics [23]. Multidrug-resistant bacteria can withstand harsh conditions by storing and passing on resistance genes within themselves and to other bacteria [24]. Wildlife’s role as natural reservoirs becomes even more critical when considering the potential routes of transmission of antibiotic-resistant strains from these organisms to humans or domesticated animals. Human activities like exploring and building on natural habitats or the wild animal trade raise the likelihood that contact will occur directly or indirectly. Such interactions increase potential instances where antibiotic resistance may transmit from wildlife populations to humans.

Wild animals very frequently share habitats with humans and domesticated animals [25], with some examples being their natural habitats (like forests and pastures), bodies of water, and even urban area. In these spaces where interaction is inevitable, there is a chance that antibiotic-resistant bacteria may exist among them, which can affect humanity both directly and indirectly through other organisms they reside within. This interaction paves the way for the transmission of drug resistance genes to other organisms, which can directly affect human health.

A variety of wildlife species cross extensive distances throughout their lifecycle, due to their migratory cycle. This results in the spreading of antibiotic-resistant bacteria into a considerable number of regions across the globe. Migratory birds are a prime example [26] as they have the capacity to transport resistant strains through significant distances and introduce them into new ecosystems. Consequently, this calls to attention why addressing antibiotic resistance needs a unified international effort, as it can lead both locally and globally to an uptick in bacterial resilience against antibiotics.

Although biodiversity within ecosystems can potentially buffer against some diseases [27] (and there have been instances of the transmission of zoonoses of bacterial origin in the past [17,18,19], and of the enterobacterial variety [20,21], like *Klebsiella* spp. is), it could also present risks relating to antibiotic resistance. The diversity of microorganisms in animal populations may contain various resistance mechanisms. It is essential to comprehend the intricacies between ecological health, biodiversity, and the likelihood of transmitting resistance genes while developing effective strategies under One Health practices.

For this, it is crucial to expand surveillance measures beyond clinical settings and include wildlife environments. Monitoring the level of antibiotic resistance within animal populations provides vital insights into prevalence rates, shifting trends over time, and how strains spread among distinct species. Integrating health concerns when devising conservation strategies can promote ecosystem stability while mitigating the risk associated with antibiotics losing their effectiveness. The microorganisms known as ESKAPE, or *Enterococcus faecalis*, *Staphylococcus aureus*, *Klebsiella pneumoniae*, *Acinetobacter *spp., *Pseudomonas aeruginosa*, and *Enterobacter* spp., are thought to be the most dangerous ones for antibiotic resistance, implying a need for a better understanding of their resistance mechanisms.

The choice to investigate antibiotic resistance specifically on *Klebsiella* spp., among the ESKAPE microorganisms, is based upon crucial considerations principally concerning its impact on animal and human well-being. A major characteristic of *Klebsiella* spp. is that it poses a clinical hazard as an aggressive pathogen among humans while also being frequently found within mucosal membranes across various wildlife species [28].

The importance of the zoonotic potential of *Klebsiella* spp. is emphasized from a One Health perspective, which dictates that human, animal, and environmental health are interdependent. Researchers have chosen to study *Klebsiella* spp. to understand antibiotic resistance patterns amongst wildlife species as well as acknowledge the likelihood for transmission between similar species and resulting impact on wild animals’ welfare.

The ability of *Klebsiella* spp. to adapt to different surroundings, such as the mucosal flora in animals, generates worries about antibiotic resistance spreading across diverse animal species. As an ESKAPE microbe, *Klebsiella pneumoniae*’s capacity for evading antibiotics intensifies its importance within research. The selection made here goes beyond just comprehending antibiotic resistance; it also pertains to the wider scope of conserving wildlife populations [29].

This study emphasizes the importance of comprehending antibiotic resistance distribution in different areas, as it provides valuable insights into its transmission dynamics. The worldwide prevalence of *Klebsiella* spp. and animal migration patterns demonstrate global implications worthy of recognition. Particularly, this research highlights how understanding wildlife migratory behaviour is crucial to gain a better grasp on the propagation of antibiotic-resistant strains.

The research highlights the detrimental impact of *Klebsiella* spp. on ecosystem dynamics, as per its opportunistic behaviour. Through an investigation into *Klebsiella* spp., researchers intend to emphasize the significance of animals as carriers for genes linked with antibiotic resistance and enhance comprehension regarding transmission rate determinants.

In essence, the reason for choosing *Klebsiella* spp. exceeds its significance in healthcare and encompasses its participation in various ecological communities, categorization among ESKAPE bacteria, and potential effects on ecosystems. This all-encompassing strategy provides a basis for examining antibiotic resistance concerning wildlife.

The 18 articles used for this review were published between 1998 and 2022, accessed between September of 2023 and October of 2023, and obtained though PubMed, ScienceDirect, and Google Scholar. The keywords used were “*Klebsiella*”, “*K. pneumoniae*”, “wildlife”, “wild animals”, and “antibiotic resistance”. The inclusion criterion was the presence of *Klebsiella* isolates in wild animals, worldwide. The method used to tabulate the study synthesis was Microsoft Excel version 16.89.1. A review protocol was not prepared.

## 2. Antibiotic Resistance

Antibiotic resistance can be classified as intrinsic, when the bacteria’s own characteristics (for example, the lack of a type of transport to the inside for the antibiotic, or an aqueous outer membrane) prevent the action of a drug, or acquired, which, as the name indicates, refers to the acquisition of a process of resistance, a process that occurs in various ways, with some of them being described below [30]. Intrinsic resistance lowers the number of antibiotics that can be used against a certain bacterial infection; for example, all Gram-negative bacteria are intrinsically resistant to glycopeptides, due to the structure of their outer membrane which prevents the entrance of the antibiotics into their periplasm. Another example is how polymyxins are ineffective against Gram-positive bacteria, because they lack the lipid this class of antibiotic binds itself to. The concept of “persistence versus resistance” is important as well: it refers to bacterial cells that are “intrinsically” resistant to an antimicrobial though they may not possess resistance against it, only because they are not currently active, i.e., dormant, so they are not able to interact with the agent [31,32]. 

The propagation of resistance genes (acquired resistance) occurs mainly through a process called horizontal gene transfer (HGT), and the bacteria’s own genes may mutate (mutational resistance), and as such, after a certain stress is applied to a group of bacteria, only those which possess a resistant mutation survive and are given the possibility of proliferation. Although mutations conferring antibiotic resistance can impose a fitness cost on bacteria, this cost is not always substantial [33]. In some cases, bacteria may manage resistance mechanisms efficiently, leading to a low energy cost in terms of growth and overall fitness [34]. The energy diverted to counter antibiotic presence may only minimally affect the bacterial physiology, especially if the resistance mechanism is well integrated into the bacterial metabolism.

Moreover, while these mutations are often selected under the pressure of antibiotic exposure, they can persist even in the absence of such pressure [34,35]. This is particularly true when the mutations are incorporated into the bacterial chromosome, where their removal would require a reversion process that may or may not occur. In other cases, resistance mechanisms are maintained on extrachromosomal elements, such as plasmids, which can exert selective pressure through plasmid stability systems or by providing other adaptive benefits to the host bacterium [34]. These factors contribute to the persistence of resistance, even when the direct selective pressure of antibiotics is not present. 

Thus, the maintenance of resistance mutations is a complex process influenced by several factors, including the efficiency of the resistance mechanism, the presence or absence of selective pressure, and the genetic context in which the mutation occurs.

There are three processes that constitute HGT: conjugation, transduction, and transformation. Conjugation is called the exchange of genes in the form of plasmids between cells, with the aid of a pilus between them. It happens in liquid media, such as water from rivers, or from water treatment plants coming from hospitals; in the soil; and in aqueous media, such as mixtures of sediments, and sludge. This process happens very often among species belonging to the Enterobacteriaceae family, which includes *Klebsiella* spp. [36]. As for mutational resistance, the actions that the mutation puts in course normally for K. pneumoniae fit into one of the following: alteration of the action site for the antibiotic; porin loss/mutation; and the upregulation of efflux pumps, in order to exit the chemical [32,37].

It is assumed that for the successful transfer and spread of resistance genes, the donor and the receiver must be in the same habitat, leading to the creation of a chain of gene exchange between bacteria. However, the presence of resistance genes does not necessarily indicate a connection with pathogenicity, as resistance mechanisms can exist in various ecological contexts unrelated to pathogenic relationships, as highlighted by recent studies [38,39]; this same chain may itself promote the evolution of those genes depending on the selective pressure they could endure during the process. For example, an inoffensive microorganism that is a natural antimicrobial producer may exert selective pressure on their peers, forcing them to evolve to maintain their lineage; in a hypothetical scenario in which the evolved microorganism offspring finds itself in animal feed, and the animal that consumes that feed is regularly treated with antimicrobial medicine in order to fend off disease, it marks another bout of selective pressure. It is possible to spread infection and resistance genes though meat that is improperly handled and then sold for human consumption [40]. If there is no selective pressure, the gene suffers no alterations and maintains its presence in the genome of the bacteria which already possess it. What this leads to is costly fitness; as with the absence of selection, the resistant bacteria will have to compete with non-resistant bacteria for resources, and as the maintenance of certain mechanisms is expensive energetically, they could be shut off and disappear from the population in favour of a less costly mechanism, especially if it is plasmid-encoded. However, there are mechanisms that have a pairing compensatory mutation, which nullifies its metabolic cost and contributes to the persistence of the gene in the bacterial population [41]. Some plasmids also contain genes that cause the lysis of the bacteria if they lose that same plasmid; resistance mechanisms that are incorporated in such structures will be maintained [42].

From a One Health perspective, this process, if not lessened through the appropriate means, poses a significant threat as entire ecosystems become compromised, and the result is undoubtedly catastrophic for the future of humankind.

**Figure 1:**
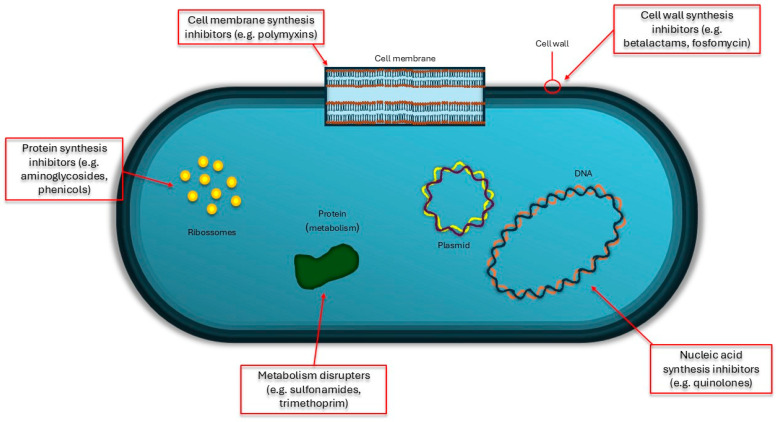
Action of the main types of antibiotics on *Klebsiella* spp.

Figure 1 depicts the action of the main antibiotics that are used on *Klebsiella* spp.

## 3. MLST

Multi-locus sequence typing (MLST) is a procedure that makes use of the sequences of internal fragments of seven housekeeping, highly conserved genes to identify different species of bacteria. This technique was borne out of the necessity of a gold standard for typing, due to the poor reproducibility of previous typing methods, in 1998, first used for *Neisseria meningitidis* [14]. MLST’s significance surpasses merely typing bacteria. Through analyzing seven housekeeping genes’ genetic sequences, MLST presents a thorough understanding of various bacterial isolates and their relations throughout evolution. This information proves to be invaluable in epidemiological research as it detects and traces clonal complexes while comprehending the dissemination patterns involved [43,44].

In the context of controlling antibiotic resistance, utilizing an MLST approach is crucial to monitor the evolution and distribution of resistant strains within populations. Due to antibiotics’ selective effects on bacterial communities forcing their adaptation, obtaining detailed knowledge about their diversity through means such as sequence analysis is important for quickly identifying mutations related to specific resistances. This provides valuable insights towards developing effective interventions that aid preventative strategies against antimicrobial treatment failure or other health risks borne from these situations.

In one of the first studies to use MLST in *Klebsiella pneumoniae*, the technique was performed with the following genes: *gapA* (glyceraldehyde 3-phosphate dehydrogenase), *infB* (translation initiation factor 2), *mdh* (malate dehydrogenase), *pgi* (phosphoglucose isomerase), phoE (phosphoporine EI), *rpoB* (beta-subunit of RNA polymerase B), and *tonB* (periplasmic energy transducer) [15].

## 4. *Klebsiella* spp.

*Klebsiella* spp. are a genus of Gram-negative bacteria, first described by Carl Friedlander in 1882, using lung samples from deceased pneumonia patients. They are frequently found in the flora of the mucosal membranes of healthy animals and humans, and in the environment (water, soil, plants). However, due to their opportunistic nature, the species that compose them can cause a wide range of serious infections and possible death in immunocompromised organisms that are exposed to them; for example, the most problematic member of this genus is undoubtedly *Klebsiella pneumoniae*. Its hypervirulent variants can cause community-acquired liver abscesses, meningitis, septic arthritis, and even systemic infection in healthy people [45], though its classic variant is plenty dangerous too, being spread worldwide and capable of grave consequences [46]. This genus is part of the Enterobacteriaceae family, which contains more pathogenic genera, such as *Escherichia*, *Salmonella*, *Serratia*, *Enterobacter*, *Yersinia*, *Raoultella*, *Citrobacter*, among others [47]. *Klebsiella* spp. can currently be divided into three groups: the *Klebsiella pneumoniae* species complex (KpSC), the *Klebsiella oxytoca* species complex (KoSC), and the remaining species, this last group comprising *K. granulomatis*, *K. aerogenes*, and *K. indica* [47]. KpSC is made up of seven phylogroups, Kp1 through 7 in the following order: *K. pneumoniae sensu stricto*, *K. quasipneumoniae* subsp. *quasipneumoniae*, *K. variicola* subsp. *variicola*, *K. quasipneumoniae* subsp. *similipneumoniae*, *K. variicola* subsp. *tropica*, *K. quasivariicola,* and *K. africana* [48,49]. KoSC consists of the following species: *K. michiganensis *(Ko1, Ko5); *K. oxytoca *(Ko2); *K. spallanzanii *(Ko3, Ko9); *K. pasteurii *(Ko4); *K. grimontii* (Ko6, Ko7); and *K. huaxiensis* (Ko8) [49,50].

This group of bacteria is rod-shaped; usually non-motile (except for *K. aerogenes*, formerly known as *Enterobacter aerogenes*); 0.3 to 1.5 µm wide by 0.5 to 5.0 µm long [51]; facultative anaerobic; and its ideal growth temperature is around 37 °C (which is why warm blooded animals make perfect habitats) and ideal pH is 7.2. When cultivated in solid media, which does not need to be especially enriched, its colonies are usually large, convex, very smooth, shiny, and mucoid, this last characteristic being caused by its K antigen on the surface of its cell wall (very apparent when in the presence of hypermucoviscous *K. pneumoniae*). As for biochemical differentiation, species can be identified through IMVIC testing, ornithine decarboxylase and lysine decarboxylase to name a few [52].

### Antibiotic Resistance Mechanisms in Klebsiella spp.

The resistance mechanisms of this genus are best exposed and studied in its type-species, *Klebsiella pneumoniae*. *K. pneumoniae*, as the type species of this genera, and the most likely to harbour and maintain antibiotic resistance mechanisms, is a good representation of the general capacity of *Klebsiella* spp. as a pathological group. 

Its main and preferred forms of resistance strategies are enzymatic inactivation of chemicals (for example, the production of beta-lactamases) [53], antibiotic target alteration [54], efflux pumps [55], biofilm formation [56], and the loss/mutation of porins [57].

In Pitout et al. [58], the authors report *K. pneumoniae* resistant to beta-lactam antibiotics due to the production of beta-lactamases such as TEM-1 and TEM-2, SHV, which exists naturally in the bacterium in question [45], and broad-spectrum beta-lactamases (ESBLs). In studies carried out by Piperaki et al. [45], it is shown to be resistant to ampicillin (AMP), carbenicillin, ticarcillin, aminoglycosides, tetracycline, trimethoprim, and sulfamethoxazole (due to the production of CTX-M), and certain carbapenems (through the production of carbapenemases, the most predominant being KPC-2 to 13, metallo-beta-lactamases such as VIM, imipenase, and NDM, and OXA. The presence of NDM-coding genes was also verified by Sidjabat et al. [59], and in Greece, 60.5% of *K. pneumoniae* isolates were resistant to carbapenems [60]. In a study conducted by Paczosa and Mecsas [46], *K. pneumoniae* was immune to monobactams such as aztreonam (TMJ) and in Paterson [61], resistance to quinolones and ceftazidime (CAZ) was observed. Due to the multiresistance of this particular species, an infection caused by the bacterium becomes something highly unlikely to cure without the use of antibiotics of last resort, such as colistin, and some carbapenems [45], something that could prove to be harmful in the near future than desired, thanks to the enormous capacity of adaptation and consequent evolution that bacteria have. However, the existence of Mobile Colistin Resistance (*mcr*) coding genes in plasmids within *K. pneumoniae* detected in France, Portugal, and China in Hassan et al. [62] and in Latin America in Quiroga et al. [63] has been proven. Resistance genes encoding class A and class D beta-lactamases have been found in the genome of *K. pneumoniae*, along with resistance genes against aminoglycosides (*aac*, *aadA*, *aph*, *strAB*, among others), colistin (as mentioned above, *mcr1*, *mcr1.2*), phenicols (*catA*, *catB*, *floR,* etc.), sulfonamides (*sul1*, *sul2*, *sul3*), tetracyclines (*tet A*, *tetB*etc), and trimethoprim (*dfr*). *K. pneumoniae* is also able to acquire mutations for the specific points of DNA gyrase (gyrA and gyrB) as well as of topoisomerase IV (parC and parE) to become resistant to quinolones, like fluoroquinolone. Another mechanism that confers quinolone resistance is the possession of DNA gyrase and topoisomerase IV protector proteins, such as Qnr [64]. As for membrane alterations, *K. pneumoniae* can create ejection pumps (e.g., AcrAB, OqxAB pumps), efflux pumps, and alter its permeability and porins (OmpK35, Omp36, OmpK26, for example) [65,66,67].

Due to the frequency of beta-lactamases found in this group’s genome, it is possible that it can play a crucial role in collective betalactams’ resistance in bacterial communities [68]. This phenomenon is characterized by the presence of resistance gene-harbouring bacteria existing in the same environment as sensitive bacteria [64]. This works due to the resistant bacteria’s ability to enzymatically inactivate the antibiotic, causing the concentration to plummet to bearable or inexistent levels. However, this can backfire if the resistance mechanism takes a toll on the resistant bacteria, freeing up more resources for the sensitive bacteria to proliferate.

**Figure 2:**
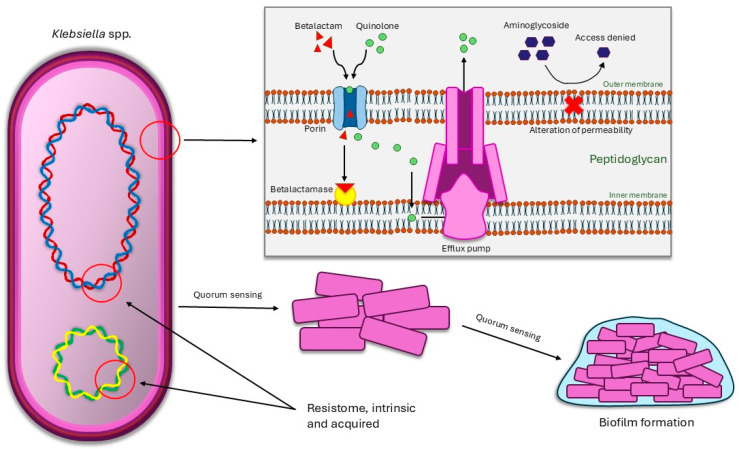
*Klebsiella* spp. mechanisms of resistance.

Another aspect of collective resistance is the formation of biofilms. Biofilms are a complex structure in which microorganisms form communities, aggregating with each other and forming glycocalyces, with the function of enhancing the survival of the “inhabitants” [69]. They can be polymicrobial (biofilm with several species of bacteria, or several associations such as bacteria–fungus), described in Kuramitsu et al. [70], which shows that dental plaque (an example of biofilm) can contain about 700 different species of bacteria, or monomicrobial (biofilm with only one species of bacteria). Polymicrobial biofilms, especially, are potentiating sites for the transfer of resistance genes between organisms [71], so they serve as a mechanism of resistance and the ease that a given bacterium has for its formation possibly demonstrates a high virulence. Biofilms also can disperse when they are fully established in the environment, through the dissociation of cells from it, followed by translocation and adhesion to the new substrate [69]. Another way that biofilms contribute to antibiotic resistance is the high density of these structures, making it so that higher concentrations of bigger molecules (like aminoglycosides) are needed to eradicate the organisms inside and that diffusion of other antimicrobials is more difficult [64]. *K. pneumoniae* tends to form biofilms, due to its capsular polysaccharides and type 1 and 3 fimbriae [72,73,74]; the first affects biofilm structure and quorum sensing while the latter promotes a steady adhesion to the chosen surface. The first in vivo description of biofilm forming *K. pneumoniae* was in 1992, when biofilms were found in the bladder and urinary fluid of patients with spinal cord injuries [75]. Since then, there have been multiple instances of biofilm forming *K. pneumoniae* and *Klebsiella* spp., in animal samples, in water, in hospital settings, in fomites, and in food [76,77,78,79,80,81,82,83,84]. A figure illustrating the main mechanisms of resistance in *Klebsiella* spp. is provided below (Figure 2).

## 5. Antibiotic Resistance in *Klebsiella* spp. Found in Wildlife

*Klebsiella* spp. are hardy organisms capable of thriving in normally harsh conditions. They are usually found in the microbiota of animals and humans, but also in fomites, soil, water, flora, and possibly air [85]. However, the number of studies detailing the prevalence, antibiotic resistance, and genetic lineages of *Klebsiella* in wildlife is low. As mentioned before, the One Health concept dictates that humankind, animals, and the environment are intricately connected, meaning that there is a constant stream of information between bacteria that live in different habitats, with some of that information being resistance genes. 

*Klebsiella* spp. represent a genus consisting of species capable of acquiring various resistance mechanisms through vertical or horizontal gene transfer, making it crucial to study how they adapt to different conditions, including wildlife environments. Several studies have identified and characterized *Klebsiella* spp. in wildlife (Table 1) such as primates, deer, bats, foxes, badgers, wolves, insects, aquatic mammals, reptiles, minks, wild boars, hogs, elephants, hares, and many species of birds [28,86,87,88,89,90,91,92,93,94,95,96,97,98,99,100,101,102]. 

In Riwu et al., in Indonesia, 129 samples of fresh deer feces were collected, and 9 total were found to be *K. pneumoniae*. Of these nine, three were proven to be multidrug resistant (all were resistant to aztreonam, tetracycline, streptomycin, and ciprofloxacin) and these same isolates also were ESBL (extended spectrum beta-lactamases) producers and possessed the *bla*_TEM_ gene [28]. Gharout et al. describes 2 samples of bat guano, collected in Algeria, out of 110 which were positive for *K. pneumoniae*, and which were both carbapenemases producers and harbourers of the *bla*_OXA-48_ gene (one of the isolates assigned to ST1878) and the *bla*_KPC-3_, *bla*_TEM-1_, and the *aac(6*′*)-Ib* genes (the other isolate belonging to ST512) [86]. Bachiri et al. details the sampling of 216 feces in Algeria (126 from barbary macaques and 90 from wild boars); 7 of the 126 were positive for ESBL-producing, *bla*_CTX-M-15_- and *bla*_TEM-1_-carrying *K. pneumoniae* and were resistant to amoxicillin, amoxicillin/clavulanic acid, ticarcillin/clavulanic acid, ceftriaxone, cefotaxime, aztreonam, ceftazidime, trimethoprim/sulfamethoxazole, gentamicin, and ciprofloxacin. A total of 10 of the 90 were found to be ESBL-producing, *bla*_CTX-M-15_- and *bla*_TEM-1_-carrying *K. pneumoniae* as well, and resistant to amoxicillin, ticarcillin/clavulanic acid, ceftriaxone, ceftazidime, cefotaxime, and aztreonam. All 17 isolates were assigned to ST584 103]. In Chiaverini et al., in Italy, 119 dead wild animals were processed, originating 131 samples. Of those, 17 (6 from wild boars, 2 from fallow deer, 2 from roe deer, 2 from red deer, 2 from European badger, 1 from magpie, 1 from red fox, 1 from wolf) were detected to be either *K. pneumoniae* or *K. quasipneumoniae*. All *K. pneumoniae* isolates were resistant to ampicillin, cloxacillin, cefazolin, and tetracycline; they also showed intermediate resistance to tobramycin and ciprofloxacin. The sole *K. quasipneumoniae* isolate showed resistance to ampicillin, cefoxitin, ceftobiprole, cloxacillin, cefazolin, ertapenem, and tetracycline. As for resistance genes, *oqxAB*, *fosA*, *bla*_SHV-1_, *bla*_SHV-11_, _blaSHV-27_, *bla*_SHV-33_, *bla*_SHV-75_, and *bla*_OKP-A-2_ were all found in the 17 isolates. One isolate (from wild boar) also carried *tetA*, *sul2*, and *strAB*. Regarding MLST analysis, the isolates show a varied pool of sequence types: ST23, ST35, ST116, ST133, ST162, ST200, ST219, ST277, ST301, ST3017, and ST4895 (*K. quasipneumoniae*). They also showed three novel types: ST*5670, ST*ca55, and ST*fc60 [88]. In Baron et al., in Senegal, 48 feces samples from 13 chimpanzees and 415 termite samples from 38 mounds were analyzed, originating 25 *K. pneumoniae* isolates from 7 chimpanzees, and 41 *K. pneumoniae* isolates from termites. Of these 66 isolates, 56 were selected to be sequenced, after which the results showed that 19 were identified as *K. quasipneumoniae* subspecies *quasipneumoniae*, 5 as *K. quasipneumoniae* subspecies *similipneumoniae*, 8 as *K. quasivariicola*, and 1 as *K. aerogenes*. Alongside this, it was found that these isolates belonged to six sequence types: ST307, ST147, ST37, ST1418, and two yet unknown STs. As for resistance genes, the following were found: *bla*_SHV–11_, *bla*_OXA–1_, *bla*_SHV–28_, *bla*_CTX–M-15_, *bla*_OXA–48_, *bla*_TEM–1B_, *bla*_KPC–2_, *blaSHV-28*, *bla*_SHV-1–2a_, *bla*_SHV–168_, *bla*_SHV–106_, *bla*_SHV–145_, and *bla*_SHV–110_ for betalactams; for aminoglycosides: *aph(6)-Id*, *aph(3*″*)-Ib*, *aac(3)-IIa*, and *aac(6*′)*Ib-cr*; for quinolones: *qnrS1*, *aac(6′)Ib-cr*, and *qnrB1*; for phenicol: *catB4*; for sulfonamides: *sul2*, *dfrA14*; for tetracycline: *tet(A)* [89]. In Du et al., in China, 10 random, pneumonia-afflicted or dead Chinese hares were selected, and 8 tested positive for *K. pneumoniae*. All eight of these isolates proved to be resistant to imipenem, meropenem, penicillin, vancomycin, polymyxin B, and ampicillin, as well as minimally sensitive to neomycin and streptomycin [90]. In Whitaker et al., in the United States of America (USA), 336 stranded marine mammals (275 California sea lions, 53 Pacific harbour seals, 3 northern elephant seals, and 5 northern fur seals) and apparently healthy wild-caught 270 California sea lions were sampled. There were also 14 samples produced before the study period, 12 from California sea lions, 1 from a Pacific harbour seal, and 1 from a harbour porpoise. All this totalled 30 *K. pneumoniae* isolates (25 from California sea lions, 4 from Pacific harbour seals, and the 1 harbour porpoise isolate): 4 from wild-caught animals, and 24 from stranded mammals [91]. In Butaye et al., in the Caribbean Island of St. Kitts and Nevis, out of 82 *K. pneumoniae* (identified by MALDI-TOF MS matrix-assisted laser desorption/ionization time-of-flight mass spectrometry) isolates, 17 belonged to vervet monkeys (*K. pneumoniae* sensu stricto), and 3 were pinpointed as *K. variicola*, again from vervet monkeys. The 17 *K. pneumoniae* vervet monkey isolates were grouped in three STs: ST137, ST37, and ST60. One vervet monkey *K. pneumoniae* isolate contained the following resistance genes: *bla*_TEM-1b_, *aph(3′)-Id*, *aph(6)*, *aac(3)-IId*, *aadA2*, *sul2*, *dfrA12*, and *tet(B)* [92]. In Lenzi et al., in Brazil, 3 *K. variicola* isolates collected from the choana of white-faced whistling ducks were grown in MacConkey agar containing polymyxin B. The isolates showed no resistance phenotype other than to polymyxin B, despite being tested against 13 other antimicrobials. The ST137 and ST167 were assigned to these isolates, and the *bla*_LEN-24_ and *bla*_LEN-13_ were detected [93]. In Brendecke et al., in Germany, 336 samples were produced from black-legged seagulls, 12 of which were determined to be ESBL-producers, though only 1 was found to be *K. pneumoniae*. This single isolate was assigned to ST290, was resistant to gentamycin, cefotaxime, ciprofloxacin, and tetracycline, and harboured the *aph(3*″*)-Ib*, *aph(6)-Id*, *bla*_CTX-M-15_, *bla*_OXA-1_, *bla*_SHV-1_, *bla*_TEM-1_, *dfrA14*, *catB3*, *fosA*, *aac(6*′*)-Ib-cr*, *oqxA*, *oqxB*, *qnrB1*, *sul1*, *tet(A)* genes [94]. In Janecko et al., in Canada, 449 samples of American crow feces were collected. In total, 41 of these were determined to be *Klebsiella* spp., and, one of the samples, named “*Klebsiella pneumoniae* ST37”, was evaluated to possess the *qnrB19* and *oqxAB* genes in 7 of its isolates [95]. In Darwich et al., in Spain, 307 wild animals included in 67 different species (birds, mammals and reptiles) were sampled. Nine of these were identified as *Klebsiella* spp., namely, *K. pneumoniae* and *K. oxytoca*. *K. oxytoca* was found in an Algerian hedgehog, harbouring the *bla*_CTX-M-3_ gene, and resistance to gentamicin, streptomycin, florfenicol, tetracycline, colistin, and trimethoprim. Four *K. pneumoniae* were discovered in European hedgehog samples, the first one being resistant to ciprofloxacin, gentamicin, streptomycin, kanamycin, tetracycline, sulfamethoxazole, and trimethoprim, with the *bla*_CMY-1_, *bla*_CMY-2_, *bla*_SHV-1_, *bla*_TEM-1_, and *bla*_CTX-M-15_ genes. The second one was found to be resistant to ciprofloxacin, nalidixic acid, gentamicin, streptomycin, kanamycin, tetracycline, sulfamethoxazole, and trimethoprim with the *bla*_SHV-11_ and *bla*_TEM-1_ genes. The third one showed resistance to ciprofloxacin, nalidixic acid, gentamicin, streptomycin, kanamycin, tetracycline, colistin, sulfamethoxazole, and trimethoprim, harbouring the *bla*_SHV-28_ gene. The fourth was resistant to ciprofloxacin, nalidixic acid, kanamycin, tetracycline, sulfamethoxazole, and trimethoprim, possessing the *bla*_SHV-12_ gene. As for birds, *K. pneumoniae* was identified twice in tawny owl samples, one harbouring the *bla*_CMY-2_ and *bla*_SHV-28_ genes. This isolate was found to be resistant to streptomycin, sulfamethoxazole, and trimethoprim. The second isolate had the *bla*_SHV-12_ and *bla*_CTX-M-15_ genes, and resistance to ciprofloxacin. This species of bacteria was found in European greenfinch as well, with the *bla*_CMY-1_ gene and resistance to ciprofloxacin, nalidixic acid, kanamycin, florfenicol, chloramphenicol, and sulfamethoxazole. Finally, *K. pneumoniae* was found in European serin, resistant to ciprofloxacin, nalidixic acid, streptomycin, kanamycin, tetracycline, sulfamethoxazole, and trimethoprim and possessing the *bla*_CMY-1_ and *bla*_SHV-28_ genes [96]. In Thornton et al., in the USA, 1123 stranded pinnipeds were sampled: 636 California sea lions, 239 harbour seals, 248 northern elephant seals. For the California sea lions, 38 isolates corresponding to *Klebsiella* spp., and 33 *K. pneumoniae* isolates were identified. For elephant seals, the numbers came to 23 *Klebsiella* spp. isolates, and 15 *K. pneumoniae* isolates. As for harbour seals, 12 *Klebsiella spp*. and 3 *K. pneumoniae* isolates were identified. All *Klebsiella* spp. specimens were sampled from superficial abscesses, wounds, ocular/urethral discharges, and umbilici. The remaining others were from various tissues post-mortem [97]. Mengistu et al., in Spain, sampled 241 wild animals (red-eared slider, American mink, and Eurasian otter). *K. pneumoniae* was found in red-eared red sliders (2), possessing the *bla*_CMY-2_ and *tetM* genes, and American minks (6), harbouring the *mcr*4 gene. *K. oxytoca* was identified in American minks (3), though they did not test positive for any of the genes used in this study [98]. In Mbehang et al., in Gabon, 125 fresh fecal samples of ground-dwelling wildlife were collected (western lowland gorilla, mandrill, collared mangabey, greater spot-nosed monkey, black colobus, duiker, genetta, waterbuck, African forest elephant, African buffalo, and red river hog). The samples produced 130 colonies, 90 of which were enterobacterial in nature. 17/90 isolates were from the *Klebsiella* genus, from the following animals and species, and with their respective resistance phenotypes: *K. oxytoca* (2) was found in western lowland gorillas, resistant to tetracycline; in African buffalo (1), resistant to amoxicillin, ticarcillin, and piperacillin; and in red river hogs (1), resistant to amoxicillin, ticarcillin, and piperacillin. *K. variicola* was found in four species of animal: western lowland gorilla (2), red river hog (1), mandrill (2), and African forest elephant (1), all sharing the same antibiotic resistances to amoxicillin, ticarcillin, and piperacillin. *K. pneumoniae* was found only in western lowland gorillas (1), along with *K. aerogenes* (4) resistant to amoxicillin, ticarcillin, and piperacillin and the *K. aerogenes* isolates were resistant to cefoxitin, too [99]. Chang et al., in the USA, sampled over 500 deceased southern sea otters and over 120 healthy individuals (for comparison). In total, 15 *K. pneumoniae* isolates, 1 *K. oxytoca* isolate, 1 *K. ornithium* isolate, and 1 *K. ozaenae* isolate were identified from various fluids, organs, wounds, and abscesses. It was also assessed that 4 of the 15 *K. pneumoniae* isolates were hypermucoviscous [100]. In McDougall et al., in Australia, 275 fecal samples from grey-headed flying foxes were collected, both from wild and captive animals undergoing rehabilitation. In total, 30 *K. pneumoniae* isolates were found, belonging to a wide variety of sequence types: ST5037, ST5033, ST1412, ST5036, and ST1017, in which all isolates were carriers of the *dfrA14* and *qnrS1* genes, and ST105, ST4919, ST661, ST5034, and ST5035, in which all isolates harboured the *bla*_SHV-110_ gene. Some other isolates carried the *bla*SHV-1 (8), *bla*_SHV-11_._v1_ (3), *bla*_SHV-27_ (8), and *bla*_SHV-229_ genes. Meanwhile, 8 *K. africana* isolates were identified and assigned to ST4939 and ST4938; all isolates carried *bla*_OKP-C-1_ variants. *K. variicola* was represented by one sole isolate, assigned to ST50372, and harbouring a variant of the *bla*_LEN-2_ gene. Six isolates of *K. oxytoca* and two isolates of *K. michiganensis* were identified as well [101]. In Dolejska et al., in Australia, 504 samples of the cloaca of silver gulls were collected, and nine isolates were identified as *K. pneumoniae*, assigned to seven STs: ST1735, with both isolates being resistant to streptomycin, sulfamethoxazole, tetracycline, sulfamethoxazole/trimethoprim, chloramphenicol, gentamicin, and meropenem; ST1736, with one isolate resistant to sulfamethoxazole, sulfamethoxazole/trimethoprim, chloramphenicol, and gentamicin; ST1734, with one isolate resistant to sulfamethoxazole, sulfamethoxazole/trimethoprim, and chloramphenicol; ST394, with one isolate resistant to streptomycin, sulfamethoxazole, sulfamethoxazole/trimethoprim, tetracycline, chloramphenicol, and gentamycin; ST1737, with two isolates: one resistant to sulfamethoxazole and gentamicin, and the other resistant to streptomycin, sulfamethoxazole, tetracycline, sulfamethoxazole, chloramphenicol, gentamycin, nalidixic acid, ciprofloxacin, and meropenem; ST1738, with one isolate resistant to sulfamethoxazole, sulfamethoxazole/trimethoprim, and gentamicin.; and ST584, with one isolate resistant to sulfamethoxazole and gentamicin [102].

This compilation of studies shows that wildlife is a possibly ideal reservoir of multidrug-resistant *Klebsiella* spp. strains, with the capacity to contaminate and be contaminated by the environment they interact with, such as water sources, scavenging, feeding, and exposure to anthropogenic materials. A trend that is visible is the frequency of resistance to betalactam antibiotics, such as penicillin, cephalosporins, carbapenems, and aztreonam, and to tetracyclines, sulfonamides, aminoglycosides, and phenicols, as well as a growing tendency to transmit colistin, quinolone resistance genes, though it must be considered that resistance genes sometimes may not be expressed, leading to an absence of a resistance phenotype [103]. In this article, a wide array of resistance genes were mentioned and the efficiency of many important classes was discussed. The classes which the genes found in this review confer resistance to are betalactams, aminoglycosides (especially streptomycin), quinolones, phosphonic acid (fosfomycin), tetracyclines, sulphonamides, phenicols, trimethoprim, and polymyxins (colistin). 

All of these classes are medically important to human medicine. The World Health Organization compiles a list (WHO MIA List) of the most important antimicrobials authorized for use on humans and animals every two years; according to the updated version of the 6th edition of this report, every class mentioned in this study ranges from “highly important” to “highest priority critically important”, with 3rd and 4th generation cephalosporins, quinolones, polymyxins, and phosphonic acid derivatives being included in this last category [104]. It is important to mention that there were carbapenem resistance genes (namely *bla*_KPC_ and *bla*_OXA_) found in wildlife, and carbapenems are only authorized for use in humans. This indicates a transmission of resistance mechanisms from human activity to nature, as does the presence of all these dangerous genes in the microbiota and tissues of wild animals; it also originates a perpetual cycle of resistance transmission and mutation that can turn back to humanity.

Some articles did not execute antibiotic susceptibility testing [92,97]; and two that did, did not mention which method was used [89,90]. However, the remaining 14 used tried and true, validated, and published susceptibility methods such as disk diffusion (Kirby–Bauer), broth microdilution, place reading systems, or plates containing a certain concentration of antibiotic [94], with the aid of official breakpoints by the European Committee on Antimicrobial Susceptibility Testing (EUCAST) and the Clinical and Laboratory Standards Institute (CLSI). Other important concepts are “clinical breakpoints” and “epidemiological cut-off values”: the former are used to predict the success that an antimicrobial may display in a clinical situation and are obtained through the analysis of many factors such as pharmacokinetic and dynamic data, several minimal inhibition concentrations (MICs), and the former results of clinical treatments and outcomes. The latter refers to one of the factors used to obtain a clinical breakpoint, namely the MIC distribution, which cannot be used to predict a clinical outcome, but can be applied in identification purposes and acquired relatively easily for any species of bacteria, given that there are enough isolates to make up a decent sample pool [105]. *Klebsiella* spp. were widely found in mammals, both aquatic and terrestrial, as well as in birds; they have been less studied in insects and reptiles, and very few times in amphibians. However, it must be considered that samples were obtained from different matrices, such as exudates, organs, feces, and dead animals as well as live (both diseased and healthy) ones, which may affect the growth and proliferation conditions of pathogenic, opportunistic bacteria. The fact that marine mammals (more isolated from human interference) were afflicted with this bacterium may mean that *Klebsiella* spp. contaminated saltwater through anthropogenic means, like wastewater draining into rivers which meet saltwater through estuaries, or directly into the sea. As cities develop increasingly, requiring more space, they encroach into natural spaces, overlapping wildlife habitat with human living. This may explain highly antimicrobial resistant strains of *Klebsiella* spp. being identified in the bodies and fecal matter of wild animals. Communal living may also explain this exchange. These studies describe 45 different sequence types, of which ST37, ST147, ST307, ST23, and ST584 were mentioned at least two times. All were found in terrestrial mammals, although ST584 and ST37 were also found in birds, and ST307 was identified in termites. This indicates a trading of genes between bacteria in different species and different environments. ST37, ST307, and ST147 have been found in clinical settings, oftentimes together [71,106,107,108,109,110,111,112,113]; ST23 is considered hypervirulent and highly resistant [114], and is found in nosocomial infections [115], capable of infecting healthy individuals [116]. ST584 is found in hospitals, too [117,118]. This indicates that *Klebsiella* spp. have few specifications they need for their host, and consequently an ease to shed and transition from habitat to habitat, leading to high rates of resistance gene transfer and acquisition.

Wildlife serves as a potential vector for antimicrobial resistance to humans and livestock though environment sharing—consider globalization and urbanization—especially when the disease agent adapts itself to many types of hosts, as is the case with *Klebsiella* spp. It is imperative that anthropological activity and its impact on the existing nature is not neglected: humans potentiate the spread of resistance mechanisms to wildlife as well. In [92], the exchange of *K. pneumoniae* with the same sequence types between humans, vervets, horses, and cats is discussed. It has also been suggested that ballast water and hull fouling may contribute to the dissemination of pathogens into other habitats [119]. It has been observed that the natural habitats that suffer the most human activity are the ones in which the highest exchange of AMR between wildlife occurs and that soil, water, and direct contact with humans are the principal hotspots of those exchanges [120].

As for different matrices/sources inside the same countries that are mentioned in Table 2, in Indonesia, *bla*_TEM_ was found in wastewater from dairy farms, dairy cows, beef cattle, broiler chickens, tilapias, and in humans [121,122,123]. In Algeria, *bla*_OXA_ and *bla*_CTX-M-15_ were found in humans [124]; *bla*_TEM_ was found in humans [125]; *bla*_KPC-3_ was found in a child (meningitis) [125]. In Italy, *bla*_SHV-27_ was found in cows and humans [126]; *bla*_SHV-11_ was found in humans [127]. In Senegal, *bla*_CTX-M-15_ was found in humans [128]. In Germany, *bla*_OXA-1_ was found in humans [129]; *catB3*, *aph(6)-Id*, *bla*_OXA-1_, *bla*_SHV-1_, *dfrA12*, *fosA*, *aac(6’)Ib-cr*, *oqxA*, *oqxB*, *qnrB1*, *sul2*, and *tetA* were found in municipal and slaughterhouse wastewaters [130]. In Spain, *bla*_CTX-M-15_, *bla*_CMY-2_, *bla*_SHV-11_, and *bla*_SHV-28_ were found in humans [131]; *bla*SHV-1 and *bla*_TEM-1_ were also found in humans [132]. In Australia, *bla*_SHV-11_, *bla*_SHV-1_, *dfrA14*, *bla*_IMP-4_, *aacA4*, and *catB3* were found in humans [133,134,135]. This demonstrates that transmission between environments exists, be it from humans to water, to cattle, or to wildlife.

When monitoring antimicrobial resistance in *Klebsiella* spp. in wildlife ecosystems, it is crucial to focus on resistance genes whose mechanisms are already well documented. Most resistance mechanisms studied in *Klebsiella* have developed under intense selective pressure in environments such as wastewater with high antibiotic concentrations, clinical settings, and patients undergoing antibiotic treatments [37,60]. Monitoring these known resistance mechanisms allows us to assess their potential risk for distribution into natural ecosystems, where they could spread among wildlife and potentially enter human and domestic animal populations.

However, it is also important to recognize that concentrating solely on known resistance mechanisms may overlook the emergence of new or cryptic resistance genes in less-studied environments like wildlife in natural ecosystems, where antibiotic pressure is typically lower; *Klebsiella* and other bacteria might develop resistance through alternative or indirect pathways that have not yet been fully understood. While monitoring known resistance genes is efficient and practical, future studies should also consider the possibility of novel resistance mechanisms evolving in wildlife, potentially driven by different environmental pressures.

In this context, balancing the surveillance of well-characterized resistance genes with exploratory investigations into new resistance mechanisms could provide a more comprehensive understanding of the risks associated with antimicrobial resistance in natural ecosystems.

Table 2 details the sequence types found in this review, highlighting the ones who are or have the potential to become pandemic, namely ST35, ST200, ST23, ST133, ST277, ST219, ST307, ST147, ST37, ST60, ST661, and ST584.

## 6. Conclusions

*Klebsiella* spp. are very well established in the microbiota of wild animals, making them a perfect reservoir of bacteria and consequent exchange of genes, be they conferring of antibiotic resistance or otherwise. This group was also shown to be well distributed across all continents and in different taxonomic classes of animals like mammals, reptiles, insects, and birds. According to the One Health concept, this means that bacteria from wildlife interacts with the bacteria from the environment and from humans, raising the potential for zoonotic disease. It is important to consider that wild animals may be important vectors of antibiotic resistance mechanisms. This review calls attention to the need for reports which track the spread of resistance genes between wildlife, the environment, humans, and vice versa, as it shows that dangerous mechanisms are currently in active transmission, and with it they are actively evolving against our current arsenal of antimicrobials; last resort antibiotics could no longer be effective in the near future, as demonstrated by the presence of resistance genes to antibiotics that are approved only for use in humans is being verified in wildlife, and consequently in the environment, opening doors for worldwide dissemination especially if found in migratory animals, as is the case in many of the situations mentioned in this study.

## Figures and Tables

**Table 1:** Animal species, location of isolation, and genetic lineages of *Klebsiella* spp. isolated worldwide. ST/CC: sequence type/clonal complex.

Animal	Location	Species	Nº of Isolates	ST/CC	Antimicrobial Resistance Genes (Prevalence)	Resistance Genes (Associated Plasmids)	Prevalence of *Klebsiella* spp.	Reference
Deer	Indonesia	*K. pneumoniae*	9	-	*bla*_TEM_ (33.33%)	-	6.97%	[28]
Bat	Algeria	*K. pneumoniae*	2	ST1878, ST512	*bla*_OXA-48_ (50%), *bla*_KPC-3_ (50%), *bla*_TEM_ (50%), *aac(6*′*)-Ib* (50%), *oqxAB* (50%)	-	1.8%	[86]
Barbary macaque	Algeria	*K. pneumoniae*	7	ST584	*bla*_CTX-M-15_ (100%), *bla*_TEM-1_ (100%)	-	5.56%	[87]
Wild boar	Algeria	*K. pneumoniae*	10	ST584	*bla*_CTX-M-15_ (100%), *bla*_TEM-1_ (100%)	-	11.1%	[87]
Wild boar	Italy	*K. pneumoniae*	6	STfc60, ST35, ST23, ST3071, ST133	*oqxAB* (100%), *bla*_SHV-75_ (16.66%), *bla*_SHV-33_ (16.66%), *bla*_SHV-11_ (33.33%), *bla*_SHV-17_ (16.66%)	*bla*_SHV-75_ (IncFIB(K)), *bla*_SHV-11_ (IncFIB(pKPHS1), IncFIB(K)(pCAV1099.114), repB(pK2044)), *bla*_SHV-33_ (IncFIB(pKPHS1), IncFIB(K)), *bla*_SHV-17_ (IncFIB(pKPHS1), IncFIB(K))	4.58%	[88]
Fallow deer	Italy	*K. pneumoniae*	2	ST301	*bla*_SHV-27_ (100%)	-	1.52%	[88]
Roe deer	Italy	*K. pneumoniae*	2	ST5670, ST2217	*bla*_SHV-1_ (50%), *bla*_SHV-11_ (50%)	-	1.52%	[88]
Red deer	Italy	*K. pneumoniae*	2	STca55, ST200	*bla*_SHV-1_ (50%), *bla*_SHV-65_ (50%)	*bla*_SHV-65_ (IncFIB(K))	1.52%	[88]
European badger	Italy	*K. quasipneumoniae*	1	ST4895	*bla*_OKP-A-2_ (100%)	*bla*_OKP-A-2_ (IncFIB(K))	0.76%	[88]
European badger	Italy	*K. pneumoniae*	1	ST162	*bla*_SHV-1_ (100%)	-	0.76%	[88]
Magpie	Italy	*K. pneumoniae*	1	ST277	*bla*_SHV-27_ (100%)	*bla*_SHV-27_ (IncFIB(K), Col(pHAD28))	0.76%	[88]
Red fox	Italy	*K. pneumoniae*	1	ST219	*bla*_SHV-1_ (100%)	*bla*_SHV-1_ (IncFIB(AP001918))	0.76%	[88]
Wolf	Italy	*K. pneumoniae*	1	ST116	*bla*_SHV-1_ (100%)		0.76%	[88]
Chimpanzee	Senegal	*K. pneumoniae*	14	ST307, ST147, ST37	*bla*_SHV-11_ (42.86%), *bla*_OXA-1_ (50%), *_bla_*_SHV-28_ (50%), *bla*_CTX-M-15_ (42.86%), *bla*_OXA-48_ (50%), *bla*_TEM-1B_ (25.71%), *bla*_KPC-2_ (42.86%), *bla*_SHV-1-2a_ (7.14%), *bla*_TEM-1_ (7.14%), *aph(6)-Id* (7.14%), *aph(3*″*)-Id* (7.14%), *qnrS1* (50%), *sul2* (50%), *tetA* (57.14), *aac(3)-IIa* (50%), *aac(6*′*)Ib-cr* (50%), *qnrB1* (50%), *catB4* (50%), *dfrA14* (50%)		29.16%	[89]
Chimpanzee	Senegal	*Klebsiella* spp.	11	-	-	-	22.92%	[89]
Termite	Senegal	*K. quasivariicola*	8	-	*bla*_LEN26_ (100%), *oqxA* (100%), *oqxB* (100%), *fosA6* (100%)	-	1.92%	[89]
Termite	Senegal	*K. quasipneumoniae*	18	-	*bla*_OKP-B-15_ (11.11%), *bla*_OKP-B-7_ (33.33%), *bla*_OKP-B-2_ (5.55%), *bla*_OKP-B-11_ (5.55%), *bla*_OKP-B-8_ (33.33%), *bla*_OKP-B-1_ (5.55%), *oqxA* (100%), *oqxB* (100%), *fosA5* (50%), *fosA6* (38.89%), *fosA3* (5.55%)	-	4.33%	[89]
Termite	Senegal	*K. aerogenes*	1	-	*oqxA* (100%)*, oqxB* (100%)*, fosA6* (100%)	-	0.24%	[89]
Termite	Senegal	*K. quasipneumoniae* subsp. *similipneumoniae*	5	-	*bla*_OKP-B-1_ (20%), *bla*_OKP-B-8_ (80%), *oqxA* (100%), *oqxB* (100%), *fosA6* (20%), *fosA5* (80%)	-	1.20%	[89]
Termite	Senegal	*K. pneumoniae*	9	ST307, ST1418	*bla*_SHV-168_ (11.11%), *bla*_TEM-1B_ (66.67%), *bla*_SHV-106_ (66.67%), *bla*_OXA-1_ (66.67%), *bla*_SHV-145_ (11.11%), *bla*_SHV-110_ (11.11%), *bla*_CTX-M-15_ (66.67%), *aac(3)-IIa* (66.67%), *aac(6*′*)Ib-a* (66.67%), *qnrB1* (66.67%), 100%), *oqxB* (100%), *fosA5* (22.22%), *fosA6* (77.78%)		2.17%	[89]
Chinese hare	China	*K. pneumoniae*	8	-	-	-	80%	[90]
Pacific harbour seal	USA	*K. pneumoniae*	4	-	-	-	7.55%	[91]
California sea lion	USA	*K. pneumoniae*	25	-	-	-	4.59%	[91]
Harbour porpoise	USA	*K. pneumoniae*	1	n.d.				[91]
Vervet monkey	St. Kitts and Nevis	*K. pneumoniae*	17	ST23, ST37, ST60, ST1102, ST2072	*bla*_SHV-40_ (64.70%), *bla*_SHV-190_ (29.41%), *bla*_SHV-26_ (5.88), *bla*_TEM-1_ (5.88%), *aph(3*″*)-Ib* (5.88%), *aph(6)-Ib* (5.88%), *aac(3)-IId* (5.88%), *aadA2* (5.88%), *oqxA* (100%), *oqxB* (100%), *fosA* (94.11%), *fosA7* (5.88%), *dfrA12* (5.88%), *tetB* (5.88%)		20.73%	[92]
Vervet monkey	St. Kitts and Nevis	*K. variicola*	3	-	-	-	4.88%	[92]
White-faced whistling duck	Brazil	*K. variicola*	3	ST137, ST167	*bla*_LEN-24_ (66.67%), *bla*_LEN-13_ (33.33%)	-	-	[93]
Black-legged seagull	Germany	*K. pneumoniae*	1	ST290	*bla*_CTX-M_, *aph(3*″*)-Ib*, *aph(6)-Id*, *bla*_OXA-1_, *bla*_SHV-1_, *bla*_TEM-1_, *dfrA12*, *catB3*, *fosA*, *aac(6*′*)-Ib-cr*, *oqxA*, *oqxB*, *qnrB1*, *sul2*, *tetA*		0.29%	[94]
American crow	Canada	*Klebsiella* spp.	41	ST37	*qnrB19* (2.44%), *oqxAB* (17%)		9.13%	[95]
Algerian hedgehog	Spain	*K. oxytoca*	1	-	*bla*_CTX-M-3_	-	0.32%	[96]
European hedgehog	Spain	*K. pneumoniae*	4	-	*bla*_CMY-1_ (25%), *_bla_*_CMY-2_ (25%), *bla*_SHV-1_ (25%), *bla*_TEM-1_ (50%), *bla*_CTX-M-15_ (25%9, *bla*_SHV-11_ (25%), *bla*_SHV-28_ (25%), *bla*_SHV-12_ (25%)	-	1.30%	[96]
Tawny owl	Spain	*K. pneumoniae*	2	-	*bla*_CMY-2_ (50%), *bla*_SHV-28_ (50%), *bla*_SHV-12_ (50%), *bla*_CTX-M-15_ (50%)	-	0.65%	[96]
European greenfinch	Spain	*K. pneumoniae*	1	-	*bla*_CMY-1_	-	0.32%	[96]
European serin	Spain	*K. pneumoniae*	1	-	*bla*_CMY-1_, *bla*_SHV-28_	-	0.32%	[96]
Elephant seal	USA	*K. pneumoniae*	15	-	-	-	6%	[97]
Elephant seal	USA	*Klebsiella* spp.	23	-	-	-	9.27%	[97]
California sea lion	USA	*K. pneumoniae*	33	-	-	-	5.18%	[97]
California sea lion	USA	*Klebsiella* spp.	38	-	-	-	5.97%	[97]
Harbour seal	USA	*K. pneumoniae*	3	-	-	-	1.25%	[97]
Harbour seal	USA	*Klebsiella* spp.	12	-	-	-	5%	[97]
Red-eared slider	Spain	*K. oxytoca*	3	-	-	-	3.30%	[98]
Red-eared slider	Spain	*K. pneumoniae*	2	-	*bla*_CMY-2_, *tetM*	-	2.20%	[98]
American mink	Spain	*K. pneumoniae*	6	-	*mcr-4*	-	31.6%	[98]
Western lowland gorilla, African forest buffalo, red river hog	Gabon	*K. oxytoca*	4	-	-	-	4.4%	[99]
Western lowland gorilla, red river hog, Mandrill, African forest elephant	Gabon	*K. variicola*	6	-	-	-	6.6%	[99]
Western lowland gorilla	Gabon	*K. pneumoniae*	1	-	-	-	1.1%	[99]
Western lowland gorilla, African forest buffalo	Gabon	*K. aerogenes*	6	-	-	-	6.6%	[99]
Southern sea otter	USA	*K. pneumoniae*	15	-	-	-	-	[100]
Southern sea otter	USA	*K. oxytoca*	1	-	-	-	-	[100]
Southern sea otter	USA	*K. ornithium*	1	-	-	-	-	[100]
Southern sea otter	USA	*K. ozaenae*	1	-	-	-	-	[100]
Pacific harbour seal	USA	*K. pneumoniae*	3	-	-	-	-	[100]
California sea lion	USA	*K. pneumoniae*	9	-	-	-	-	[100]
Grey-headed flying fox	Australia	*K. pneumoniae*	30	ST5037, ST5033, ST1412, ST5036, ST1017, ST105, ST4919, ST661, ST5034, ST5035	*bla*_SHV-110_ (50%), *bla*_SHV-1_ (26.7%), *bla*_SHV-11_._v1_ (10%), *bla*_SHV-27_ (26.7%), *bla*_SHV-299_ (3.33%), *dfrA14* (10%), *qnrS1* (10%)		10.9%	[101]
Grey-headed flying fox	Australia	*K. africana*	8	ST4939, ST4938	*bla*_OKP-C-1_ (100%)	-	2.9%	[101]
Grey-headed flying fox	Australia	*K. variicola*	1	ST50372	*bla*_LEN-2_	-	0.36%	[101]
Grey-headed flying fox	Australia	*K. oxytoca*	6	-	-	-	2.18%	[101]
Grey-headed flying fox	Australia	*K. michiganensis*	2	-	-	-	0.73%	[101]
Silver gull	Australia	*K. pneumoniae*	9	ST1735, ST1736, ST1734, ST394, ST1737, ST1738, ST584	*bla*_IMP-26_ (22.2%), *bla*_IMP-4_ (77.78%), *qacG* (100%), *aacA4* (100%), *catB3* (100%)		1.78%	[102]

**Table 2:** Sequence types mentioned in this review per country and whether they were found in other sources.

Sequence Type	Country	Other Sources	References	Other Countries	References
ST1878	Algeria	Humans, water	[136,137]	Australia, Romania	[101,138]
ST512	Algeria	Humans, animals	[87,139]	Spain, Italy, Poland, Taiwan	[140,141,142]
ST584	Algeria	First report in Algeria	[87]	Slovakia	[117]
STfc60	Italy	Novel ST	[88]	Not found	Not found
ST5670	Italy	Novel ST	[88]	Not found	Not found
STca55	Italy	Novel ST	[88]	Not found	Not found
ST35	Italy	Humans (newborns), urban wastewater	[143,144,145]	France, Tunisia, Spain, Yemen, Denmark	[146,147,148,149]
ST23	Italy	Humans	[126,150]	Taiwan, South Korea, India	[151,152]
ST3071	Italy	Novel ST	[88]	Not found	Not found
ST133	Italy	Not found	Not found	Ghana, Japan, Brazil	[153,154,155]
ST301	Italy	Not found	Not found	Tunisia	[156]
ST5670	Italy	Novel ST	[88]	Not found	Not found
ST2217	Italy	Not found	Not found	China	[157]
STca55	Italy	Novel ST	[88]	Not found	Not found
ST200	Italy	Not found	Not found	India, Ghana, China, Norway	[158,159,160,161]
ST4895	Italy	Not found	Not found	Not found	Not found
ST162	Italy	Not found	Not found	Slovenia	[162]
ST277	Italy	Human	[163]	England, Portugal, China	[164,165,166]
ST219	Italy	Human	[167]	Algeria, Türkiye, Russia, Germany, Lebanon	[168,169,170,171]
ST116	Italy	Not found	Not found	China	[172]
ST307	Senegal	Not found	Not found	Switzerland, Germany, Columbia, South Korea, Italy, Vietnam, South Africa	[58,173,174,175,176]
ST147	Senegal	Not found	Not found	Ghana, Greece, Slovenia, Tunisia, Algeria, Kenya, Nigeria	[177]
ST37	Senegal	Human	[178]	French West Indies, India, China	[179,180,181]
ST23	St. Kitts and Nevis	Not found	Not found	Brazil, China	[182,183]
ST37	St. Kitts and Nevis	Not found	Not found	French West Indies, India, China	[179,180,181]
ST60	St. Kitts and Nevis	Not found	Not found	Brazil, France, Bangladesh	[184,185,186]
ST1102	St. Kitts and Nevis	Not found	Not found	Japan	[187]
ST2072	St. Kitts and Nevis	Not found	Not found	Not found	Not found
ST137	Brazil	Not found	Not found	Spain	[188]
ST167	Brazil	Not found	Not found	China	[189]
ST90	Germany	Human	[190]	France,	[191]
ST37	Canada	Not found	Not found	French West Indies, India, China	[179,180,181]
ST5037	Australia	Not found	Not found	Not found	Not found
ST5033	Australia	Not found	Not found	Not found	Not found
ST1412	Australia	Not found	Not found	Algeria, Tunisia	[192,193]
ST5036	Australia	Not found	Not found	Not found	Not found
ST1017	Australia	Not found	Not found	Germany, China	[193,194]
ST105	Australia	Not found	Not found	China	[195]
ST4919	Australia	Not found	Not found	China	[196]
ST661	Australia	Not found	Not found	Thailand, USA, UK	[197,198,199]
ST5034	Australia	Not found	Not found	Not found	Not found
ST5035	Australia	Not found	Not found	Not found	Not found
ST4939	Australia	Not found	Not found	Not found	Not found
ST4938	Australia	Not found	Not found	Not found	Not found
ST50372	Australia	Not found	Not found	Not found	Not found
ST1735	Australia	Not found	Not found	Not found	Not found
ST1736	Australia	Not found	Not found	Not found	Not found
ST394	Australia	Not found	Not found	Brazil	[200]
ST1737	Australia	Not found	Not found	Not found	Not found
ST1738	Australia	Not found	Not found	China	[201]
ST584	Australia	Not found	Not found	Slovakia, Malaysia, Algeria	[87,117,202]
